# Purinergic Signaling Pathway in Human Olfactory Neuronal Precursor Cells

**DOI:** 10.1155/2019/2728786

**Published:** 2019-04-02

**Authors:** Héctor Solís-Chagoyán, Edgar Flores-Soto, Marcela Valdés-Tovar, Montserrat G. Cercós, Eduardo Calixto, Luis M. Montaño, Carlos Barajas-López, Bettina Sommer, Arnoldo Aquino-Gálvez, Citlali Trueta, Gloria A. Benítez-King

**Affiliations:** ^1^Instituto Nacional de Psiquiatría Ramón de la Fuente Muñiz, Laboratorio de Neurofarmacología, Calzada México-Xochimilco 101, San Lorenzo Huipulco, CP 14370 Ciudad de México, Mexico; ^2^Universidad Nacional Autónoma de México, Departamento de Farmacología, Facultad de Medicina, CP 04510 Ciudad de México, Mexico; ^3^Instituto Nacional de Psiquiatría Ramón de la Fuente Muñiz, Departamento de Neurofisiología, Calzada México-Xochimilco 101, San Lorenzo Huipulco, CP 14370 Ciudad de México, Mexico; ^4^Instituto Nacional de Psiquiatría Ramón de la Fuente Muñiz, Departamento de Neurobiología, Ciudad de México, Mexico; ^5^Instituto Potosino de Investigación Científica y Tecnológica, Camino a la Presa San José 2055, Col. Lomas 4ª Sección, CP 78216 San Luis Potosí, Mexico; ^6^Instituto Nacional de Enfermedades Respiratorias, Departamento de Investigación en Hiperreactividad Bronquial, CP 14080 Ciudad de México, Mexico; ^7^Instituto Nacional de Enfermedades Respiratorias, Laboratorio de Oncología Biomédica, CP 14080 Ciudad de México, Mexico

## Abstract

Extracellular ATP and trophic factors released by exocytosis modulate *in vivo* proliferation, migration, and differentiation in multipotent stem cells (MpSC); however, the purinoceptors mediating this signaling remain uncharacterized in stem cells derived from the human olfactory epithelium (hOE). Our aim was to determine the purinergic pathway in isolated human olfactory neuronal precursor cells (hONPC) that exhibit MpSC features. Cloning by limiting dilution from a hOE heterogeneous primary culture was performed to obtain a culture predominantly constituted by hONPC. Effectiveness of cloning to isolate MpSC-like precursors was corroborated through immunodetection of specific protein markers and by functional criteria such as self-renewal, proliferation capability, and excitability of differentiated progeny. P2 receptor expression in hONPC was determined by Western blot, and the role of these purinoceptors in the ATP-induced exocytosis and changes in cytosolic Ca^2+^ ([Ca^2+^]_i_) were evaluated using the fluorescent indicators FM1-43 and Fura-2 AM, respectively. The clonal culture was enriched with SOX2 and OCT3/4 transcription factors; additionally, the proportion of nestin-immunopositive cells, the proliferation capability, and functionality of differentiated progeny remained unaltered through the long-term clonal culture. hONPC expressed P2X receptor subtypes 1, 3-5, and 7, as well as P2Y2, 4, 6, and 11; ATP induced both exocytosis and a transient [Ca^2+^]_i_ increase predominantly by activation of metabotropic P2Y receptors. Results demonstrated for the first time that ex vivo*-*expressed functional P2 receptors in MpSC-like hONPC regulate exocytosis and Ca^2+^ signaling. This purinergic-triggered release of biochemical messengers to the extracellular milieu might be involved in the paracrine signaling among hOE cells.

## 1. Introduction

In many species including humans, neurogenic process in the central nervous system (CNS) and the olfactory epithelium (OE) prevails in adulthood [1, 2]. This allows the replacement of dead cells [3] to preserve structural and functional features of both the CNS and OE by the integration of de novo specialized cells in preestablished neuronal circuits [4, 5]. This process requires the proliferation and migration of multipotent stem cells (MpSC) that are capable of self-renewal, and their progeny differentiates into neuronal or glial lineages [6].

Stem cells can be dissociated from neurogenic niches to develop primary cultures in a monolayer [7]. However, cellular composition of these tissue samples is heterogeneous, including undifferentiated MpSC (these cells possess the potency to differentiate into diverse cellular lineages and are capable of indefinite self-renewal in culture) and mitotically active committed progenitors (they have limited potency and finite culture lifetime), as well as immature and mature cells. Hence, to study the physiology of MpSC in culture, an additional experimental procedure is required to specifically isolate them from heterogeneous primary cultures. In this regard, cloning through limiting dilution grants the acquisition of a culture composed predominantly by multipotent precursors, driven from the multiple division of a single MpSC [8]. The effectiveness of cloning is usually corroborated through functional criteria and detection of specific protein markers expressed by these stem cells [9–11].

The culture of stem cells obtained from the human olfactory epithelium (hOE) had enabled the study of diverse cellular processes that are dependent on Ca^2+^ signaling in neuronal cells, for instance, the olfactory transduction in sensory neurons [12], axonogenesis [13, 14], exocytosis triggered by depolarization of the plasma membrane [15], and microtubule organization [16]. Therefore, this cell culture could be an ex vivo suitable model to study the Ca^2+^-dependent mechanisms underlying proliferation [17], migration [18], or differentiation [19] in neuronal precursor cells. These three cellular processes have been associated with the activation of the purinergic signaling pathway by adenosine 5′-triphosphate (ATP) in rodent embryonic and adult stem cells [20–24]. However, the expressed P2 receptor subtypes and the role played by these purinoceptors remain virtually uncharacterized in undifferentiated precursors derived from human neurogenic structures.

It is well-known that ATP participates in many metabolic processes, as it is a molecule that contains high-energy bonds. Besides, this nucleotide has also been recognized as an extracellular messenger that mediates paracrine signaling by activating membrane purinoceptors expressed in neuronal and nonneuronal tissues, such as the OE, brain, kidney, and liver, among others [25]. P2 receptors have been classified as P2X and P2Y [26, 27]. Ionotropic P2X receptors are ligand-gated nonselective cationic channels and subunits P2X1 to 7 have been described. Activated P2X receptors are predominantly permeable to Ca^2+^ and Na^+^, allowing the increase of these cations in the cytosolic space. Meanwhile, metabotropic P2Y receptors are heptahelical G-protein-coupled receptors proteins with eight different subtypes: P2Y1, 2, 4, 6, 11, 12, 13, and 14. ATP binding to P2Y receptors leads to either phospholipase C (PLC) activation and synthesis of inositol 1,4,5-trisphosphate (IP_3_) to induce Ca^2+^ release from intracellular stores or modulation of adenylate cyclase and consequent changes in concentration of cyclic adenosine monophosphate (cAMP) and in the activity of protein kinase A (PKA) [28].

P2 receptor activation by ATP in mesenchymal stem cells has been associated with exocytosis of trophic factors, such as fibroblast growth factor 2 (FGF2), brain-derived neurotrophic factor (BDNF), nerve growth factor (NGF), vascular endothelial growth factor (VEGF), or the neuropeptide Y [29–31]. By these means, ATP might regulate autocrine or paracrine signals in this cellular type. Moreover, these mesenchymal precursors express several P2 receptor subtypes, further pointing out that purinergic signaling is involved in the modulation of different cellular processes [18]. Since key neurogenic phenomena are modulated by purinoceptors, our aim was to characterize the P2 receptors' expression in ex vivo human olfactory neuronal precursor cells (hONPC) with MpSC-like features and in addition, by evaluating exocytosis, to explore whether this Ca^2+^-dependent purinergic signaling pathway might play a role in paracrine communication between cells from the hOE.

## 2. Material and Methods

### 2.1. Primary Culture of Cells from Human Olfactory Epithelium

**Figure 1:**
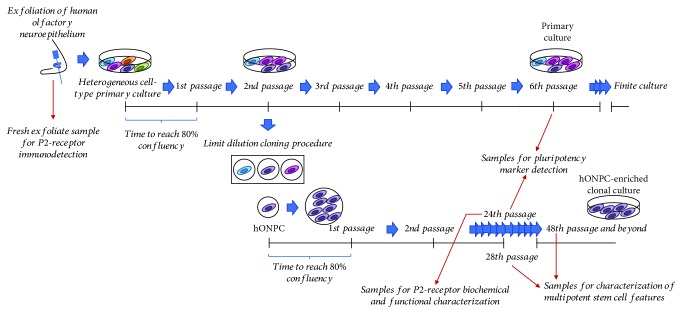
Timeline for cell culture procedures. Cells were obtained by exfoliation of the human olfactory epithelium and cultured in DMEM/F12 medium supplemented with 10% FBS, 2 mM glutamine, and 1% streptomycin-penicillin, at 37°C with 5% CO_2_. A clonal culture was obtained through the limiting dilution procedure. Red arrows indicate the precise passages from where samples were taken for experiments. hONPC: human olfactory neuronal precursor cell.

This study was conducted in compliance with the Declaration of Helsinki for research involving humans, with the understanding and written consent of the subject. The experimental protocol was previously approved by the Institutional Ethical Committee (INPRFM IC 092010.0). The sample donor was a healthy 54-year-old female without personal or familial history of neuropsychiatric disorders. Cells of heterogeneous primary cultures were obtained from the hOE by exfoliation of the nasal cavity (Figure 1), following the protocol described in detail [32]. Briefly, mechanically dissociated cells were cultured in Dulbecco's modified Eagle medium/nutrient mixture F-12 (DMEM/F12), supplemented with 10% fetal bovine serum (FBS), 2 mM L-glutamine, and 1% streptomycin-penicillin at 37°C with 5% CO_2_. Subcultures were cryopreserved in liquid nitrogen adding 8% DMSO to supplemented medium. All culture reagents were from Gibco® and Thermo Fisher Scientific (Waltham, MA, USA); the rest of the reagents were from Sigma-Aldrich (St. Louis, MO, USA) unless otherwise stated. Also, a fresh exfoliate sample was taken from the same donor for immunodetection of P2 receptors (Figure 1).

### 2.2. Cloning of Olfactory Neuronal Precursor Cells from the Primary Culture

Cloning was performed to isolate hONPC from cryopreserved heterogeneous primary cultures as described in detail previously [33]. This procedure grants the establishment of a homogeneous culture and an increase in robustness of responses restricted to the hONPC population. Briefly, thawed cells of the primary culture in passage 2 were cultured in 25 cm^2^ flasks. At 80% confluency, cells were detached with 0.25% trypsin-EDTA in PBS (in mM): 137 NaCl, 2.7 KCl, 10 Na_2_HPO_4_, and 2 KH_2_PO_4_; pH 7.3. Limiting dilution was performed reseeding one cell per well in a 96-well plate (Figure 1). Single-attached proliferating cells were cultured with supplemented medium until progeny reached confluency. At this point, the detachment protocol with trypsin was used to expand the clonal cultures in 25 or 75 cm^2^ flasks. A clonal culture was cryopreserved in liquid nitrogen in different passages and used for the experiments. First, effectiveness of cloning to isolate MpSC-like precursors was corroborated; afterwards, the purinergic pathway in these hONPC was studied.

### 2.3. Specific Pluripotency Marker Detection

To corroborate cloning effectiveness, specific protein markers expressed by pluri- or multipotent precursors were detected by using the Proteome Profiler™ Human Pluripotent Stem Cell Array Kit (R&D Systems; Minneapolis, MN, USA). It consists of nitrocellulose membranes with duplicate spots of 15 different preadsorbed antibodies. Cloned cells in passage 24 and cells of the heterogeneous primary culture in passage 6 were seeded in 75 cm^2^ flasks (Figure 1). At 80% confluence, cells were scraped with the kit's lysis buffer and processed following the instructions given by the supplier. Briefly, total protein was determined [34] to add 150 *μ*g protein by membrane. Protein markers were detected by chemiluminescence with a ChemiDoc™ MP Imaging System and analyzed with the Image Lab™ software (Bio-Rad Laboratories, Hercules, CA, USA). The protein profiles were determined in two membranes by culture; therefore, densitometric data of 4 spots by protein were compared between cultures with a Mann-Whitney *U* test.

### 2.4. Detection of Specific Cell-Type Markers

To detect some additional specific protein markers in hONPC, clonal culture at passages 28 and 48 (Figure 1) was processed for indirect immunofluorescence staining. Fixed cloned cells with 4% paraformaldehyde/PBS were permeabilized with 0.1% Tween-20/PBS. Nonspecific sites were blocked with 3% bovine serum albumin (BSA)/PBS. All primary antibodies were incubated overnight at 4°C. Proteins stained were as follows: nestin to detect precursor cells (1 : 200, Millipore MAB5326) [11, 35], vimentin to stain hOE-derived precursors (1 : 100, Invitrogen 18-0052) [36], olfactory marker protein (OMP) to detect spontaneously differentiated olfactory sensory neurons (OSN) (1 : 100, Abcam ab62144) [37], and neuronal enolase to stain mature neurons (1 : 250, Millipore MAB324). Fluorochrome-conjugated secondary antibodies were incubated for 2 h at room temperature (FITC- or TRITC-conjugated anti-species-IgG (H+L)) (Jackson ImmunoResearch), and nuclei were stained with DAPI (200 nM). Coverslips were mounted with PVA-DABCO® medium, and preparations were observed with an epifluorescence Nikon Eclipse TE2000 microscope (Tokyo, Japan) and a 40x objective (NA 1.30). Images were acquired with a Nikon DS-2MV camera and the Nikon NIS-Elements AR software. The percentage of stained cells was determined by counting the total number of nuclei and the number of cells stained with each antibody in six random-selected fields by triplicate. The primary antibody incubation was omitted for negative controls. Results were transformed with the arcsin function, and a paired Student *t* test was carried out to compare them between passages.

### 2.5. Olfactory Neuronal Precursor Cell Proliferation Capability

Proliferation levels of cloned hONPC were evaluated by quantifying incorporated BrdU through an ELISA kit (Roche, Bromo-2′-deoxy-uridine Labeling and Detection Kit III), following the manufacturer's instructions. Briefly, thawed cloned cells in passages 28 and 48 were seeded in a 96-well plate, in a density of 5000 cells/well, and cultured for 3 days; then, BrdU was added for 1 h. Absorbance was read at 405 and 490 nm with a Benchmark Microplate Reader (BioRad) to calculate the absorbance ratio by quadruplicate. Proliferation in early and late passages was compared through a Student *t* test.

### 2.6. Mature Olfactory Sensory Neuron Functionality

hOE precursors can spontaneously differentiate into OSN under culture. Mature OSN show distinctive morphology and evoke voltage-activated Ca^2+^ currents (VACC) [12]. Thus, we measured VACC to confirm the identity of these mature neurons but principally to challenge the persistence of functionality in differentiated hONPC's progeny in a long-term clonal culture. Electrophysiological recording of VACC was performed by a patch clamp with the whole-cell configuration [38] following the conditions described in detail by Solís-Chagoyán et al. [12, 16]. Briefly, cloned hONPC in passages 28 and 48 were cultured with supplemented medium for 4 days. OSN were selected for recordings through morphological criteria as previously described; i.e., OSN are characterized by a round or ellipsoidal soma from which a dendrite with a knob at its end is projected [12, 16, 32]. Cells were perfused at room temperature with a solution in which Ca^2+^ was replaced by Ba^2+^ as the charge carrier. This extracellular solution contained (in mM) 136 NaCl, 6 CsCl, 5 BaCl_2_, 10 HEPES, 11 D-glucose, and 0.1 niflumic acid, pH 7.4 adjusted with CsOH. Pipette microelectrodes of 4–6 MΩ were filled with a solution containing (in mM) 130 CsCl, 5 MgCl_2_, 10 HEPES, 10 EGTA, 3 ATP-disodium, and 1 GTP sodium salt, pH adjusted to 7.3 with CsOH. Voltage clamp was controlled with an amplifier (Axopatch 200A, Axon Instruments). The electrical signals were filtered at 1–5 kHz and digitized at 10 kHz (Digidata 1200, Axon Instruments); currents were analyzed with the software pCLAMP (version 9.0, Axon Instruments). VACC were evoked by depolarizing steps (duration 500 ms, at 1 Hz) ranging from –60 to +50 mV, in steps of 10 mV from a holding potential of –70 mV. Cell capacitance was measured throughout all the experiments. Current peaks were measured from 8 cells per passage and a current-voltage (*I-V*) relationship was plotted. Data were compared between passages by a Student *t* test.

### 2.7. P2 Receptor Detection by Western Blot

Expression of P2 receptors in ex vivo hONPC was determined by Western blot. Cloned precursors (passage 24) were cultured in 75 cm^2^ flasks and scraped with RIPA lysis buffer containing (in mM) 50 Tris (pH 7.5), 0.5 EDTA, 0.25% deoxycholate, 1% Nonidet™ P40, 1 PMSF, 0.5 sodium orthovanadate, and 20 *μ*g/mL protease inhibitors: aprotinin, leupeptin, and pepstatin. Cellular lysis was performed by sonication (3 pulses, 40 Hz, 30 s), and total protein concentration was determined by Lowry's method. Separated proteins (10 *μ*g) by SDS-PAGE electrophoresis were transferred into PVDF or nitrocellulose (NITRO) membranes. Nonspecific sites were blocked with a TBS commercial buffer (Odyssey, LI-COR) for 30 min. Primary antibodies in blocking buffer were incubated overnight at 4°C. The primary antibodies were as follows: P2X1 (PVDF; Abcam ab81122; 1 : 1500), P2X2 (PVDF; Abcam ab10266; 1 : 500), P2X3 (PVDF; Abcam ab90905; 1 : 1000), P2X4 (PVDF; Alomone APR-002; 1 : 1000), P2X5 (NITRO; Alomone APR-027; 1 : 500), P2X7 (NITRO; Alomone APR-004; 1 : 500), P2Y2 (PVDF; Alomone APR-010; 1 : 1000), P2Y4 (PVDF; Alomone APR-006; 1 : 1000), P2Y6 (NITRO; Alomone APR-011; 1 : 500), and P2Y11 (NITRO; Santa Cruz sc-98600; 1 : 500). Secondary biotinylated antibodies in 0.05% Tween-20 in TBS were incubated for 1 h: anti-rabbit-IgG (P2X1 1 : 400,000; P2X2 1 : 300,000; P2X3 80,000; P2X4 1 : 70,000; P2X5 1 : 70,000; P2X7 1 : 70,000; P2Y2 1 : 50,000; P2Y4 1 : 50,000; and P2Y6 1 : 70,000) and anti-goat-IgG (P2Y11 1 : 20,000). All membranes were incubated with peroxidase-streptavidin (Bio-Rad, 1 : 70,000) and chemiluminescent substrate (Millipore). Chemiluminescence was detected with a ChemiDoc™ MP System and the Image Lab™ software (Bio-Rad Laboratories, Hercules, CA, USA). Antibody specificity controls were assessed either without incubation with the primary antibody (P2X3) or by preadsorption of primary antibodies with the corresponding blocking peptides (control antigens for APR-027 (P2X5) and for APR-010 (P2Y2); Alomone), for those P2 receptors that revealed more than one chemiluminescent band.

Detection of P2Y2 and P2Y4 was performed in a protein extract obtained from a fresh exfoliate sample of the hOE. This fresh exfoliate sample was lysed, sonicated, separated in a 10% polyacrylamide gel (7 *μ*g), transferred to PVDF membrane, and blocked as described for the cloned precursors. Primary P2Y2 and P2Y4 antibodies (1 : 700) in blocking buffer were incubated overnight at 4°C. After 1 h incubation with peroxidase anti-rabbit IgG antibody (1 : 30,000), membranes were processed for chemiluminescent signal detection as described before.

### 2.8. ATP-Induced Exocytosis in Olfactory Neuronal Precursor Cells

Exocytosis induced by ATP in cloned precursors (passage 24) was evaluated by detection of the fluorescent styryl dye FM1-43. hONPC plated on glass-bottom cell-imaging dishes (Eppendorf) were cultured for 3 days. Afterwards, the medium was replaced by 1.35 mL physiological Hank's solution (137 mM NaCl, 5.36 mM KCl, 1.26 mM CaCl_2_·2H_2_O, 1.09 mM MgCl_2_·6H_2_O, 0.81 mM MgSO_4_·7H_2_O, 4.2 mM NaHCO_3_, 0.44 mM KH_2_PO_4_, 1.33 mM Na_2_HPO_4_, and 5.5 mM D-glucose), containing 24 *μ*M FM1-43 (Molecular Probes, Eugene, OR, USA). Emitted fluorescence was detected with a Nikon Eclipse TE2000 microscope and a 40x objective (NA 1.30) using an excitation filter passing 460–500 nm wavelengths, an emission filter passing 510 nm, and a CCD camera (Nikon DS-Ri2). Image sequences (640 × 480 pixels) were acquired at a rate of 0.5 frames/s during 482 s using the NIS-Elements AR version 2.3 (Nikon) software. Preparations were observed 10 minutes after addition of FM1-43 to reach a stable incorporation of the dye on the plasma membranes. Basal fluorescence was established for 120 s, and then 150 *μ*L of a 10X ATP stock (final concentration 100 *μ*M; final volume 1.5 mL) was added manually with a micropipette to stimulate exocytosis in hONPC. Vehicle responses were determined by adding regular Hank's solution without ATP. To determine the purinergic receptors' involvement in ATP-stimulated exocytosis, two strategies were followed: first, the P2 receptor antagonist suramin (100 *μ*M) was added 5 min before ATP and second, the P2Y agonist uridine 5′-triphosphate (UTP; 100 *μ*M) was added instead of ATP. Suramin was prepared as a 100X stock solution and UTP as a 10X stock solution so that volumes added to reach the desired concentrations were 15 *μ*L and 150 *μ*L, respectively (final volume 1.5 mL in all cases). Fluorescence was measured from selected regions of interest (ROI) drawn around single cells. To calculate the fluorescence changes relative to the basal fluorescence, the average intensity in the 40 frames before stimulation (*F*_0_) was subtracted from the intensity of that ROI at each time (*F*(*t*)). The difference was divided by *F*_0_, to generate Δ*F*/*F*_0_. For simplicity, this normalization is referred to as *dF*/*F* throughout the text. Response's amplitude and velocity (analyzed from the first derivative of the fluorescence increase) from 8 different dishes (5 cells/dish) per ATP, UTP, and suramin+ATP groups were measured and compared through a one-way ANOVA.

### 2.9. [Ca^2+^]_i_ Measurement in Olfactory Neuronal Precursor Cells

To evaluate [Ca^2+^]_i_ changes induced by ATP in cloned precursors, cells (passage 24) were plated in round coverslips coated with rat collagen and cultured for 2 days. Then, cells were loaded for 1 h (at 37°C and 5% CO_2_) with 2.5 *μ*M Fura-2 AM in low Ca^2+^ (0.1 mM)_._ Precursors were perfused in a heated chamber (at 37°C) mounted on an inverted Nikon Diaphot 200 microscope (Tokyo, Japan) at a rate of 2–2.5 mL/min with a Krebs solution bubbled with carbogen (in mM): 118 NaCl, 25 NaHCO_3_, 4.6 KCl, 1.2 KH_2_PO_4_, 1.2 MgSO_4_, 11 D-glucose, and 2 CaCl_2_. Loaded hONPC were subjected to alternating pulses of 340/380 nm excitation light, and emitted fluorescence was detected at 510 nm using a microphotometer (model D-104), from Photon Technology International (PTI, Princeton, NJ, USA). Fluorescence was measured at a rate of 0.5 s, and [Ca^2+^]_i_ was calculated according to the Grynkiewicz formula [39] considering the parameters described in detail by Solís-Chagoyán et al. [12]. Data were stored and analyzed using data acquisition and analysis software (Felix version 1.21; PTI) [40]. To evaluate Ca^2+^ response to ATP, cells were perfused with 100 *μ*M ATP to stimulate the purinergic pathway. To pharmacologically determine if P2Y receptors were involved in this response, a receptor's agonist (UTP, 100 *μ*M) or an antagonist (Reactive Blue 2 (RB2), 100 *μ*M) plus ATP was used. To further confirm the involvement of a metabotropic pathway, the G-protein uncoupler N-ethylmaleimide (NEM, 100 *μ*M) plus ATP was added [41, 42]. Participation of P2X receptors in this response was determined by perfusing the agonist *α*-*β*-methylene ATP (100 *μ*M) and the antagonist pyridoxalphosphate-6-azophenyl-2′,4′-disulfonic acid (PPADS, 30 *μ*M) plus ATP and also by perfusing cells with Krebs solution containing ATP but without Ca^2+^. To determine the contribution of P2Y and P2X receptors to the global response, two stimuli were applied with an interstimulus period of 15 min. The first response was to ATP; in the second, only the agonists or the antagonists plus ATP were perfused. The difference between the maximal amplitude of each response minus the mean basal [Ca^2+^]_i_ was calculated from 5 cells by treatment and compared through a Student *t* test between stimuli.

## 3. Results

### 3.1. Olfactory Neuronal Precursor Cells Expressed Specific Protein Markers of Multipotent Stem Cells

**Figure 2:**
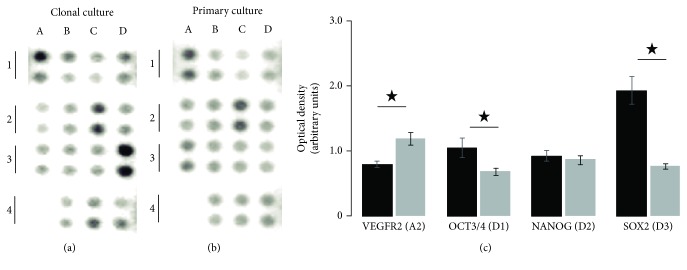
Proteome profile of human multipotent stem cell markers. Neuronal precursor cells were isolated from a hOE heterogeneous primary culture by cloning. Cloned cells at passage 24 (a) and primary culture cells at passage 6 (b) were lysed, and 150 *μ*g of total protein was added to each membrane of the antibody array. The optical density of immunopositive spots was quantified by quadruplicate. The proteins corresponding to duplicate spots of the array were as follows: Snail (A1), VEGF R2 (A2), HCG (A3), and negative control (A4); SOX17 (B1), OTX2 (B2), TP63 (B3), and Goosecoid (B4); *α*-Fetoprotein (C1), GATA-4 (C2), HNF-3*β* (C3), and PDX-1 (C4); and OCT3/4 (D1), NANOG (D2), SOX2 (D3), and E-cadherin (D4). (c) depicts data comparison for some relevant proteins. Data represent the media ± SEM. Statistical analysis was carried out with a Mann-Whitney *U* test. ^∗^*p* < 0.05.

To corroborate that hONPC with MpSC-like traits were effectively isolated from the primary culture and predominated in the clonal culture, we looked for protein markers specifically expressed by pluri- and multipotent precursors with an antibody array. Results were compared between the clonal culture and the heterogeneous primary culture (Figure 2). The proteome profile obtained by this procedure showed that transcription factors such as SOX2, NANOG, and OCT3/4 were detected in both samples. However, the SOX2 and OCT3/4 transcription factors showed significantly higher optical density in the clonal culture (SOX2 primary culture: 0.77 ± 0.04, clonal: 1.94 ± 0.21 and OCT3/4 primary culture: 0.69 ± 0.05, clonal: 1.05 ± 0.15) (Figures 2(a) and 2(c)). Meanwhile, the optical density corresponding to type 2 receptor of VEGF was higher in the primary culture (primary: 1.19 ± 0.09; clonal: 0.8 ± 0.05) (Figures 2(b) and 2(c)). These data suggest that human olfactory neuronal stem cells are present in both primary and clonal culture but enriched in the latter. The clonal culture showed key proteomic features closely related to those of MpSC.

### 3.2. Specific Functional Features Prevail Indefinitely in Olfactory Neuronal Precursor Cells and Their Mature Progeny

**Figure 3:**
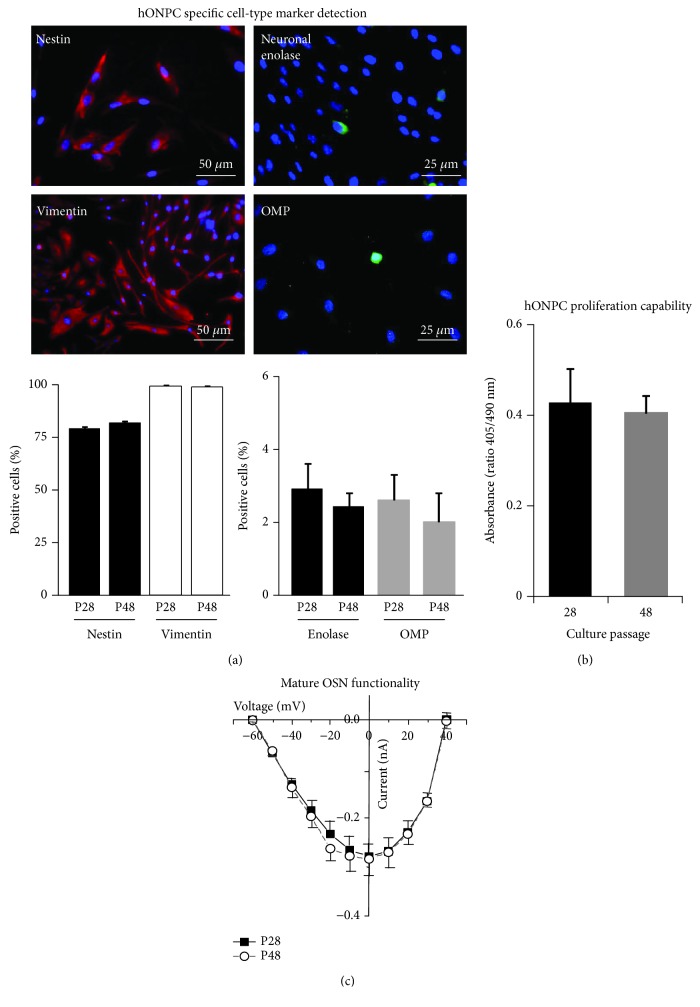
Characterization of the clonal culture by detection of specific protein markers and functional evaluation of precursor cells. Cloned cells from passages 28 (P28) and 48 (P48) were cultured and processed. In (a), the upper panel shows representative images from P28 cells stained by immunofluorescence. Nuclei were detected with DAPI (blue staining). Lower panels illustrate comparisons of immunopositive cells between passages (*n* = 18 fields per protein tested). (b) shows the proliferation level assessed through BrdU incorporation into DNA, measured through an ELISA assay (*n* = 4 wells per passage). (c) shows the evaluation of functionality of mature olfactory sensory neurons (OSN) through recording of voltage-activated Ca^2+^ currents (VACC) by a patch clamp (8 cells per passage). Data represent the media ± SEM and were compared by Student *t* test.

Self-renewal property, a key functional characteristic observed in MpSC cultures, was assessed by comparing the proportion of nestin-immunopositive cells in the clonal culture between distant passages (Figure 3(a)). Nestin is an intermediate filament expressed specifically by precursor cells. In this regard, 80% of cells were stained using the primary antibody against this protein and no statistical differences were found when comparing passages 28 and 48 (P28: 79.2 ± 3.2%; P48: 81.9 ± 2.6%) (Figure 3(a)). Moreover, the intermediate filament protein vimentin that is expressed in neuronal and nonneuronal cells from the OE, was immunodetected to corroborate the olfactory neuroepithelial origin of hONPC (Figure 3(a)). Vimentin (+) cells were almost 100% in both passages, and comparison showed no statistical differences (P28: 97.1 ± 3.1%; P48: 96.3 ± 2.4%) (Figure 3(a)). These data indicate that precursor cells predominated in the clonal culture from the hOE.

The differentiation capability of cloned precursors was determined by detection of OMP (that is expressed specifically by mature OSN) (P28: 2.6 ± 0.7%; P48: 2.1 ± 0.8%) and the neuronal enolase protein (P28: 2.7 ± 0.7%; P48: 2.4 ± 0.4%) (Figure 3(a)). These markers were detected in ~2% of cells, and no statistical differences were found for any of them when comparing the percentage of positive cells between the culture passages 28 and 48 (Figure 3(a)). These results indicate that a small proportion of hONPC progeny spontaneously differentiated into OSN under our culture conditions and that the differentiation capability persisted in a long-term culture.

As was mentioned before, self-renewal in a long-term culture is one important functional feature of MpSC [8, 43]; also, progeny of these proliferating cells can differentiate into diverse mature phenotypes. Therefore, we determined the proliferation capability of the cloned precursors by BrdU incorporation into DNA. Furthermore, the functionality of spontaneously differentiated OSN was evaluated by VACC electrophysiological recording (this function plays a key role in the olfactory transduction).  Figure 3(b) shows that cloned precursors cultured at distant passages (28 or 48) incorporated the thymidine analog BrdU at similar levels, and no statistical differences were detected when comparing this function between passages (P28: 0.43 ± 0.07; P48: 0.41 ± 0.04). Regarding the VACC functionality showed in Figure 3(c), statistical differences between those passages were not found, neither in the current peak evoked by depolarization pulses (P28: −0.28 ± 0.07 nA; P48: −0.27 ± 0.05 nA) and the reversal potential (P28: 39.4 ± 2.1 mV; P48: 39.8 ± 2.3 mV) nor in the potential at which half of the maximal current was reached (P28: −34.2 ± 1.2 mV; P48: −35.4 ± 2 mV). Altogether, these results suggest that both the proliferation capability of hONPC (undifferentiated cells) and excitability of spontaneously differentiated OSN (specialized cells) were unaltered indefinitely in the clonal culture.

### 3.3. Olfactory Neuronal Precursor Cells Express P2 Receptors

**Figure 4:**
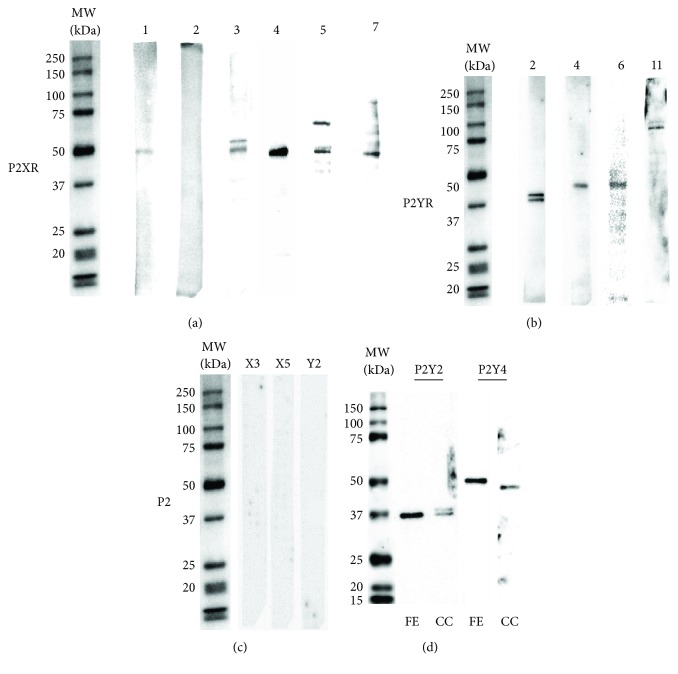
Detection of P2 receptors in the clonal culture of the hOE. Cloned cells were scraped with a RIPA buffer, and cell lysates were assayed by Western blot to immunodetect P2 receptor subtypes. Representative chemiluminescent bands corresponding to P2X receptors are shown in (a) and to P2Y receptors in (b). (c) shows antibody specificity controls assessed by omission of primary antibody (P2X3) or by preadsorption of anti-P2X5 and anti-P2Y2 with the corresponding blocking peptides. The molecular weights of sample bands were corroborated using biotinylated molecular weight standards. (d) shows immunodetection of P2Y2 and P2Y4 receptors in both the fresh exfoliate (FE) sample and the clonal culture (CC) from the hOE.

Purinergic receptors are a family of proteins found in a broad spectrum of mammalian tissues. Its activation *in vivo* induces proliferation and differentiation of stem cells from the OE and CNS in rodents. Thus, we explored if the ex vivo preparation of cloned precursors retains the ability to express P2 receptors.  Figure 4(a) shows that except for subtype 2, all other P2X receptor subtypes (P2X1, 3, 4, 5, and 7) were found. The chemiluminescent bands detected using the specific antibodies corresponded with proteins around 50 kDa, as expected. Double bands were found with the P2X3 and P2X5 antibodies. Moreover, P2Y2, 4, 6, and 11 receptor subtypes were detected in bands at molecular weights between 40 and 45 kDa, except for P2Y11 that was found at approximately 90 kDa (Figure 4(b)); a doublet appeared for P2Y2.  Figure 4(c) shows antibody specificity controls performed for P2 receptors that displayed more than one band, i.e., P2X3, X5, and Y2. No chemiluminescent signal was detected for any of these proteins. These data show that both ionotropic and metabotropic P2 receptors were expressed in the *ex vivo* preparation of cloned hONPC. To corroborate the expression of P2Y2 and P2Y4 in the human olfactory epithelium in situ, we detected these proteins in a fresh exfoliate (FE) sample (Figure 4(d)). Single chemiluminescent bands in the expected molecular weights were found for both receptors in this sample.

### 3.4. Purinergic-Dependent Exocytosis in Olfactory Neuronal Precursor Cells

**Figure 5:**
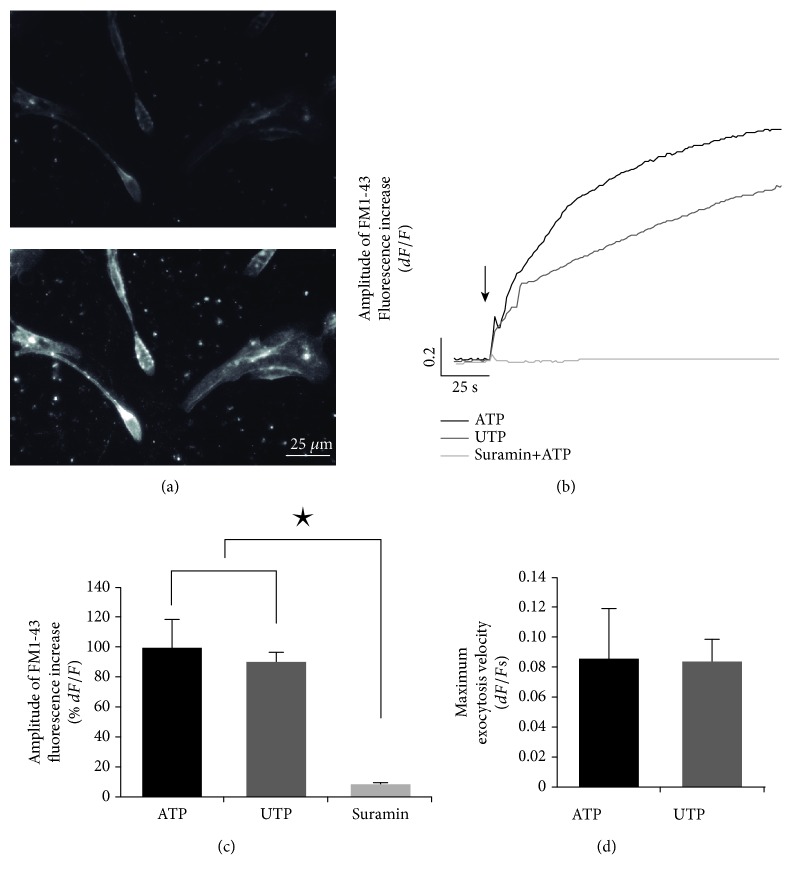
Purinergic-dependent exocytosis in olfactory neuronal precursor cells. Cloned cells were cultured for 3 days, and exocytosis was evaluated using the fluorescent indicator FM1-43. (a) shows human olfactory neuronal precursor cells (hONPC) before stimulus (up) and visible fluorescence augmentation after ATP stimulation (down). (b) depicts the fluorescence mean response kinetics after applying ATP, UTP (P2Y receptor agonist), or suramin (P2 receptor antagonist) plus ATP. The arrow shows the time when stimulus was applied. (c) and (d) show bar graphs comparing responses' amplitude and velocity, respectively. Data represent the media ± SEM and were compared through a one-way ANOVA and a post hoc Tukey test (8 dishes per treatment, 5 cells/dish). ^∗^*p* < 0.05.

In mesenchymal and CNS-derived stem cells, purinoceptors stimulate exocytosis of trophic factors and this process is involved in paracrine signaling. Thus, we studied the ATP-induced exocytosis in cloned hONPC. Stimulation of these precursors with ATP enhanced the FM1-43 fluorescence intensity (Figures 5(a) and 5(b)). In addition, UTP (P2Y receptor agonist) induced a similar response than ATP (Figures 5(b) and 5(c)), and no statistical differences in maximal amplitude (ATP: 100 ± 19.1%; UTP: 90.2 ± 7.3%) or velocity (ATP: 0.09 ± 0.03; UTP: 0.08 ± 0.02) were detected between these groups (Figure 5(c) and 5(d)). However, in cells incubated with suramin (P2Y and P2X receptor nonselective antagonist) plus ATP, the response was completely blocked (suramin+ATP: 7 ± 1.7%) (Figure 5(c)). These results suggest that the purinoceptors expressed in the ex vivo preparation of hONPC were functional, and they induced exocytosis principally by the activation of P2Y receptors.

### 3.5. ATP Induced [Ca^2+^]_i_ Increase in Olfactory Neuronal Precursor Cells

**Figure 6:**
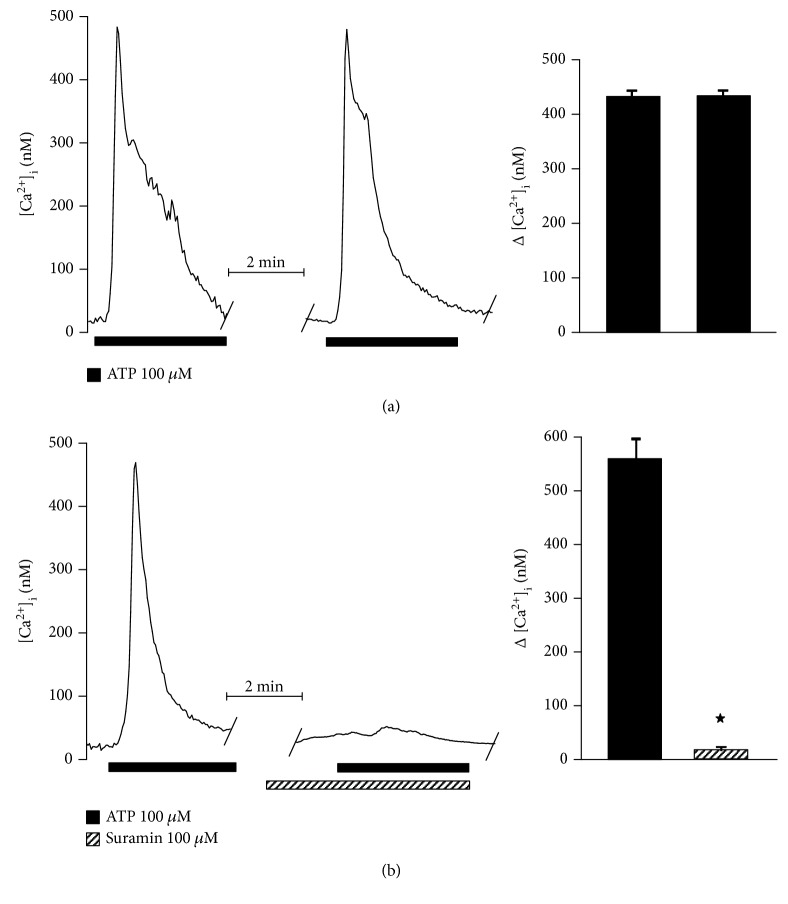
ATP-induced increase in cytosolic Ca^2+^ is mediated by P2 receptor activation in precursors from the hOE. Clonal cells were cultured for 3 days to study the change in cytosolic Ca^2+^ by microfluorometry using the fluorescent indicator Fura-2 AM. Two pulses of ATP were applied with an interstimulus interval of 15 min to determine the response reproducibility (a) (*n* = 5). (b) shows that the Ca^2+^ increase induced by ATP was blocked by suramin (*n* = 5). Data represent the media ± SEM and were compared by a Student *t* test. ^∗^*p* < 0.05.

It is well-known that activation of purinoceptors is followed by an increase in [Ca^2+^]_i_. Therefore, we corroborated the functionality of the expressed P2 receptors by microfluorometry, detecting cytosolic free Ca^2+^ with the indicator Fura-2 AM. hONPC perfusion with ATP induced a transient increase in [Ca^2+^]_i_ (Figure 6(a)). When two stimuli of ATP were given, the increase in [Ca^2+^]_i_ was similar between stimuli and no statistical differences were detected (first stimulus increase: 436.8 ± 11.5 nM; second stimulus: 437.2 ± 10.4 nM) (Figure 6(a)). In addition, suramin completely blocked the response to ATP (ATP: 551.3 ± 20.4 nM; suramin: 12.5 ± 3.1 nM) (Figure 6(b)), indicating that the [Ca^2+^]_i_ increase was induced by P2 receptor activation.

**Figure 7:**
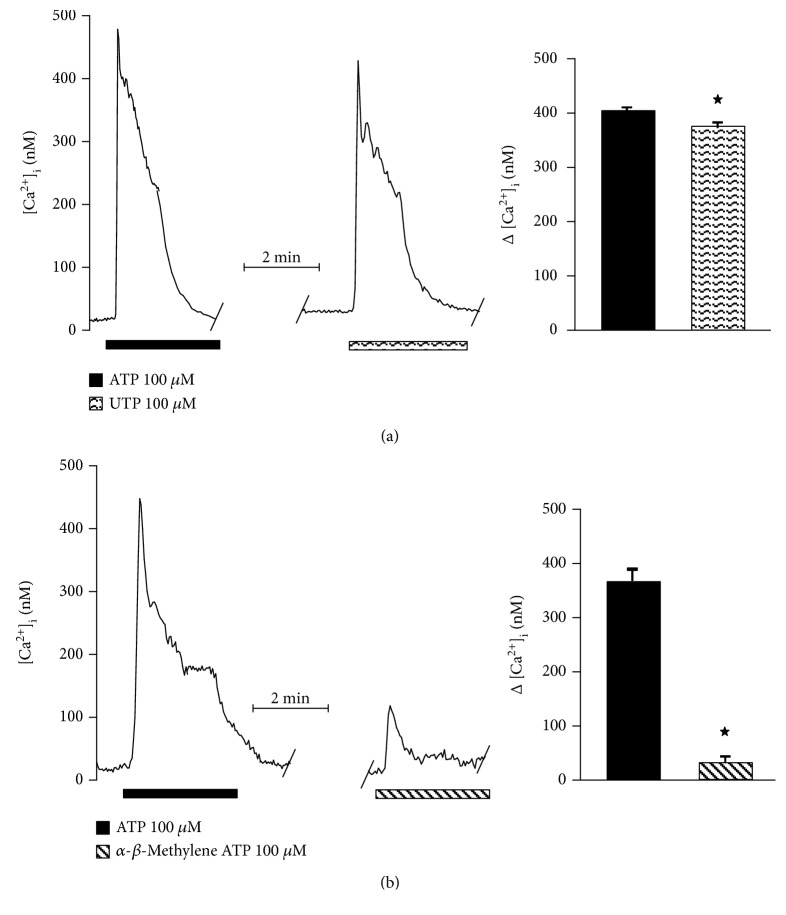
Participation of P2Y and P2X receptors in the cytosolic Ca^2+^ increase induced by ATP in olfactory neuronal precursors cells. To determine the specific relative contribution of either P2Y or P2X receptors to the enhancement of cytosolic Ca^2+^ induced by ATP, specific agonists were applied at the second stimulus and compared with the response to ATP. The response to UTP (P2Y agonist) reached 90% of the ATP response (a) (*n* = 5), whereas the P2X agonist *α*-*β*-methylene ATP only induced 10% of the ATP response (b) (*n* = 5 cells). Data represent the media ± SEM and were compared through a Student *t* test. ^∗^*p* < 0.05.

**Figure 8:**
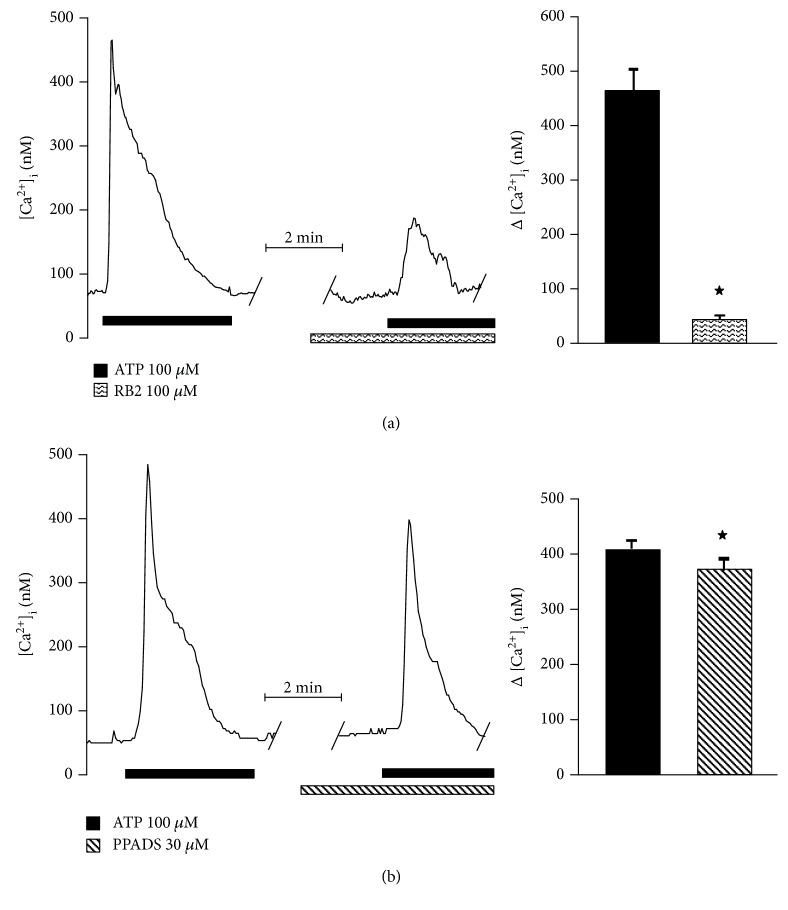
Cytosolic Ca^2+^ increase induced by P2Y and P2X receptor activation is blocked by their antagonist in precursors from the hOE. Cell perfusion with RB2 (P2Y receptors antagonist) blocked 90% of the ATP response (*n* = 5) (a), whereas PPADS (P2X receptors antagonist) blocked only 10% (*n* = 5) (b). Data represent the media ± SEM and were compared by a Student *t* test. ^∗^*p* < 0.05.

Therefore, to further determine the involvement of either P2Y or P2X in this response, specific agonists of P2Y (UTP) or P2X (*α*-*β*-methylene ATP) were used. The former induced a [Ca^2+^]_i_ increase equivalent to 91% of the ATP-evoked response (ATP: 414.6 ± 3.8 nM; UTP: 378.6 ± 6.9 nM) (Figure 7(a)), whereas *α*-*β*-methylene ATP only augmented [Ca^2+^]_i_ in 9.6% (ATP: 370.6 ± 12 nM; *α*-*β*-methylene ATP: 35.6 ± 6.4 nM) (Figure 7(b)). Additionally, the perfusion of RB2 (P2Y receptor antagonist) blocked 88% of the response to ATP (ATP: 461.8 ± 45.3 nM; RB2: 54.6 ± 4.6 nM) (Figure 8(a)), while PPADS (a selective P2X antagonist) blocked the response to ATP by only 8.5% (ATP: 407.6 ± 17.3 nM; PPADS: 373.2 ± 18.4 nM) (Figure 8(b)).

**Figure 9:**
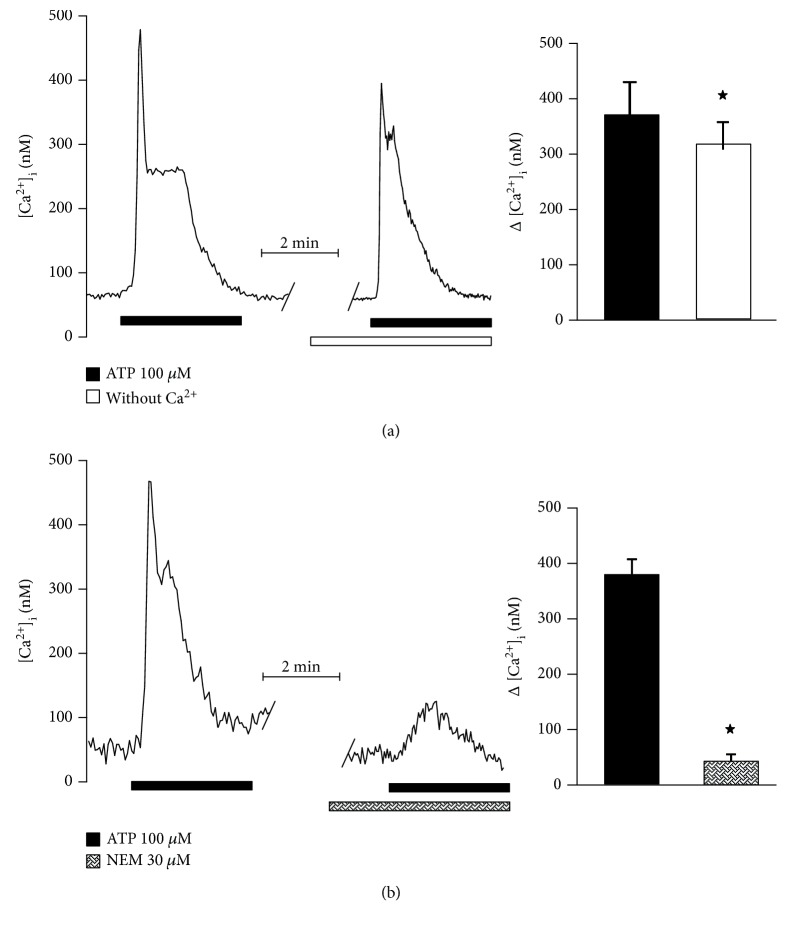
Contribution of the ionotropic or the metabotropic pathway to the purinoceptor-mediated response to ATP in precursor cells of the hOE. Ionotropic P2X receptors induce an extracellular Ca^2+^ influx when stimulated, while G-protein coupled P2Y receptors induce release of Ca^2+^ from the intracellular stores through IP3 stimulation. The contribution of Ca^2+^ influx through P2X receptors in hOE precursors was determined by perfusing cells with a Ca^2+^-free solution (a) (*n* = 5) and stimulating them with ATP. The metabotropic P2Y pathway was blocked using NEM, a G-protein uncoupler (b) (*n* = 5). The former diminished the response to ATP by 10%, and NEM blocked the response by 90%. Data represent the media ± SEM and were compared with a Student *t* test. ^∗^*p* < 0.05.

ATP might augment [Ca^2+^]_i_ by two pathways, by depletion of intracellular Ca^2+^ stores through activation of metabotropic P2Y receptors, or by influx of extracellular Ca^2+^ upon activation of ionotropic P2X receptors. To explore the participation of these mechanisms, hONPC were perfused with ATP in a Ca^2+^-free solution to discard Ca^2+^ influx. In this case, the response to ATP reached 84% (ATP: 382.5 ± 65.1 nM; Ca^2+^-free: 321.1 ± 54.7 nM) (Figure 9(a)); conceivably, this ATP response does not rely on extracellular Ca^2+^ influx through P2X receptors. Contrastingly, when the trimeric G-protein was uncoupled with NEM, the response to ATP was reduced by 88% (ATP: 378.8 ± 25.9 nM; NEM: 45.9 ± 12.3 nM) (Figure 9(b)). These results point out that the metabotropic response mediated by G-proteins and Ca^2+^ efflux from intracellular stores was predominant in the global response to ATP in hONPC.

## 4. Discussion

In this study, we found for the first time that *ex vivo* cultured hONPC that exhibit MpSC-like features expressed functional purinergic P2 receptors coupled to Ca^2+^ signaling. This purinergic pathway induced exocytosis and a transient increase in [Ca^2+^]_i_, predominantly by the activation of metabotropic P2Y receptors. In CNS neurogenic niches, trophic factors released by exocytosis play a key role in the regulation of proliferation, migration, or differentiation of MpSC. Hence, the ATP-mediated exocytosis in hONPC might be involved in paracrine signals to modulate these processes in the hOE.

MpSC are distributed in the OE of humans and rodents, and they have been isolated and cultured by different methodologies [44, 45]. Recently, it was shown that neuronal precursors obtained by exfoliation of the nasal cavity can form neurospheres, and cells derived from them retain in culture the self-renewal capability as well as the clonogenic and differentiation abilities [46]. MpSC possess proteomic and functional traits that are useful to corroborate its adequate isolation; for instance, they specifically express SOX2, OCT3/4, and NANOG [9, 10, 47], and transfection of plasmids or viral vector-driven expression of genes codifying these 3 transcription factors has been successfully used to reprogram somatic cells to acquire traits of pluripotent precursors [48–54]. In this regard, in the OE of Sprague-Dawley rats *in vivo*, SOX2 expression is restricted to MpSC and this transcription factor is absent in committed progenitors and immature or mature OSN [55].

Therefore, to corroborate that limiting dilution allowed the isolation of a single MpSC-like hONPC to expand a clonal culture, protein markers' expression and functional traits were evaluated. Regarding the markers, SOX2, OCT3/4, and NANOG transcription factors were detected in both the primary and the clonal cultures, even though the clonal culture had significantly higher levels of SOX2 and OCT3/4; in contrast, NANOG presented a similar level in both cultures. Additionally, the VEGF-receptor type 2 (VEGFR2) was augmented in the primary culture. This receptor regulates the migration of committed progenitor cells in rodents [56, 57] as well as in hOE-derived progenitors, acting upon focal adhesions [58]. VEGFA (which binds to R2 receptor type) might also induce differentiation into endothelial cells [59]. In the present work, the noteworthy differences in the expression level of the mentioned proteins between cultures suggest that hONPC with MpSC features predominate in the culture established by cloning; meanwhile, committed progenitor cells were more abundant in the primary culture.

In addition to the protein profile, we further complemented the characterization of our clonal culture testing functional traits. In *in situ* as well as in long-term MpSC cultures, some key functions are preserved such as self-renewal property, division capability, and functionality of differentiated progeny [8, 43]. Thus, we proved that viability of cloned precursors was sustained at least until passage 60, as opposed to mitotically active cells from the heterogeneous primary culture that underwent evident senescence at passage 9 or 10 [32]. To further support this finding, it was confirmed that the proportion of precursor cells detected by nestin immunostaining predominated in the clonal culture and it remained unchanged until late passages, suggesting that hONPC's population was preserved indefinitely in a long-term culture by their own proliferation, as it occurs in MpSC monolayer cultures [8].

Moreover, a small proportion of cells in the clonal culture spontaneously differentiated into mature OSN as confirmed by OMP staining, and differentiation capability of hONPC was sustained until late passages. This allowed us to determine whether the functionality of hONPC's progeny remained unchanged indefinitely by evaluation of VACC. On the one hand, OSN acquire VACC along their differentiation process under culture [12]; hence, parameters of these ionic currents might reflect if the expression level of channels and their modulation were preserved. On the other hand, VACC are a key element involved in the chemical to electrical olfactory transduction [60]; therefore, the properties of the evoked response of VACC might suggest if mature OSN would act properly upon an odorant stimulation [12]. As expected, this specialized function also persisted beyond the late 48th passage, suggesting that cellular integrity was preserved indefinitely in de novo specialized OSN. Functional results concerning proliferation of undifferentiated cells and excitability of differentiated cells also support that cloning was effective to isolate hONPC with MpSC-like traits and these precursors predominated in the expanded clonal culture.

In this study, we characterized the purinergic pathway in a clone derived from the hOE. *Ex vivo* hONPC expressed a broad range of ionotropic (P2X1, 3, 4, 5, and 7) and metabotropic (P2Y2, 4, 6, and 11) receptors. This finding is similar to the expression of P2 receptors in other stem cells such as mesenchymal precursors, where a broad variety of purinoceptors participate in the purinergic signaling [18]. In our work, P2X3, P2X5, and P2Y2 receptors were detected as doublets. This fact can be related to posttranslational modifications such as protein glycosylation, where covalent bonds with functional groups might modify the relative mobility of proteins in polyacrylamide gels, as reported in other studies concerning these P2 receptors [61, 62]. Furthermore, the band corresponding to P2Y11 had a higher molecular weight than expected. In this regard, it has been described that genes codifying P2Y11 and the Peter Pan protein (PPAN) can be transcribed as a single mRNA and translated into an unfunctional complex with a molecular weight of about 100 kDa [63]. These results indicate that ex vivo cloned hONPC expressed a diverse variety of ionotropic P2X and metabotropic P2Y receptors.

To further characterize the functionality of purinoceptors in the *ex vivo* preparation of hONPC, we assessed exocytosis by FM1-43 dye. Stimulated hONPC showed similar kinetic responses and maximal fluorescence intensity to either ATP or UTP. Upon dye immersion into the membrane lipid bilayer and excitation with the adequate wavelength, this indicator fluoresces [64]. Therefore, an increase in fluorescence intensity is seen as an enhancement in the membrane surface due to vesicles' fusion [64]. In this regard, the increase in FM1-43 fluorescence dependent on the purinergic stimulus could be due to an exocytotic process. Since UTP is a pyrimidine nucleotide that binds specifically to P2Y2 and P2Y4 receptors [65], these novel data suggest that in hONPC, ATP triggered exocytosis mainly by activation of P2Y2 and/or P2Y4 receptors. The specific activation of P2 receptors was corroborated because changes in fluorescence were completely ablated by the antagonist suramin.

Feasibly, exocytosis implies release of the vesicular content to the extracellular compartment, but the biochemical identity of such content remains undefined. The hypothesis that P2 receptors activated exocytosis is supported by the fact that progenitor cells from a hOE primary culture can release cytokines or trophic factors stored in vesicles such as interleukin-6 (IL-6) [66], neurotrophin-4 (NT-4) [67], and epidermal growth factor (EGF) [68]. Furthermore, the release of such trophic factors occurs upon activation of the purinergic signaling pathway in mesenchymal stem cells [69, 70]. Thus, it is highly possible that there is an ATP-dependent release of trophic factors in hONPC, and if this occurs, this paracrine mechanism might modulate the communication between hONPC and other cell types such as glia or vascular cells in the hOE. In support of this, in neurogenic niches *in vivo* from the rat CNS, a paracrine signal dependent on released trophic factors from MpSC and glial cells self-regulates the appropriate functioning of the niche [71]. Clearly, this issue warrants further research.

Another well-studied function of ATP in stem cells is the induction of changes in [Ca^2+^]_i_ [18]. Our data showed that both P2Y and P2X receptors were activated by ATP; however, P2Y receptors were defined as the main contributors to this Ca^2+^ increase, since 90% of the global [Ca^2+^]_i_ augmentation was induced by UTP. As mentioned, metabotropic P2Y2 and P2Y4 receptors (both detected in this work by Western blot) are activated by purine and pyrimidine nucleotides [65]. Particularly, P2Y2 receptors have been associated with induction of proliferation in MpSC from the subventricular zone in mice [72] and human melanoma precursors [20]. In this sense, EGF and FGF2 mitogens incubated with UTP induce proliferation in human mesencephalic neural stem/precursor cells [24, 73]. On the other hand, P2X receptors also participated in the ATP-induced [Ca^2+^]_i_ increase; nevertheless, its participation in exocytosis remains to be elucidated. These ionotropic receptors could be involved in other cellular functions; for instance, the P2X7 receptor has been associated with apoptosis regulation in cells from different tissues [74]. This suggest that purinergic signaling might be involved in the hOE homeostasis, maintaining the balance between proliferation and apoptosis since this epithelium is continuously injured by environmental insults [75]. Although the specific role of each P2 receptor in hONPC remains unexplored, this work reports for the first time the characterization of the purinergic signaling pathway in stem cells that possess multipotent traits isolated from the hOE.

Evidence concerning physiology of neuronal precursors obtained from healthy humans could constitute the basis to perform future comparison studies with hONPC isolated from patients diagnosed with neuropsychiatric disorders such as schizophrenia. In this regard, anomalies in the purinergic signaling pathway have been detected in patients diagnosed with this illness [76, 77]. In addition, postmortem studies have indicated dysfunction regarding neurogenic processes regulated by P2 receptors such as proliferation, migration, and differentiation of precursor cells in schizophrenic patients [32, 78, 79]. Olfactory mucosa biopsies obtained from healthy controls and patients with neuropsychiatric diseases have allowed dissociation and culture of precursors that form neurospheres. From these, MpSC have been isolated by cloning and these cell lines maintain specific neuropsychiatric disease-associated alterations such as gene and protein expression and functionality [80]. Therefore, hONPC could be a model to study physiopathology of mental disorders including alterations in the neurogenic processes modulated by the purinergic signaling. Furthermore, these anomalies might even become useful biomarkers contributing to an accurate diagnosis of neuropsychiatric illnesses.

## 5. Conclusions

The long-term cell culture obtained by cloning was composed predominantly by hONPC that showed proteomic and functional traits closely related to MpSC. These stem cells were confirmed to have originated from the olfactory neuroepithelium because they expressed vimentin and OMP. The preparation of ex vivo hONPC expressed a broad variety of P2 receptors. This purinergic pathway induced both an exocytotic process and a transient increase in [Ca^2+^]_i_ mainly through activation of metabotropic P2Y receptors. Hence, in the human olfactory neuroepithelium, ATP might function as a chemical messenger modulating the paracrine signaling among the different cell types that need to be coordinated in this neurogenic structure. Our results indicate that the culture of hOE-derived neuronal precursor cells is a suitable model to study the Ca^2+^-dependent purinergic signaling pathway and the involvement of P2 receptors in proliferation, migration, or differentiation in human multipotent precursor cells.

## Data Availability

The data used to support the findings of this study are available from the corresponding authors upon request.
